# Identification of obstruent contrasts by children with and without phonological disorders

**DOI:** 10.1590/2317-1782/e20240086en

**Published:** 2025-03-03

**Authors:** Mayara Ferreira de Assis, Elissa Barbi Mouro Pagliari Cremasco, Isabella Rodrigues Domingues, Larissa Cristina Berti

**Affiliations:** 1 Universidade Estadual Paulista “Júlio de Mesquita Filho” – UNESP - São José do Rio Preto (SP), Brasil.; 2 Universidade Estadual Paulista “Júlio de Mesquita Filho” – UNESP - Marília (SP), Brasil.

**Keywords:** Perception, Evaluation, Phonological Disorder, Child Development, Speech Intelligibility

## Abstract

**Purpose:**

(1) To compare auditory-perceptual accuracy and reaction time in children with and without phonological disorders for identifying the contrast of obstruents, and (2) to verify whether there is an effect of the phonetic class (stops vs. fricatives) on the accuracy, reaction time and error pattern.

**Methods:**

Sixty-two children (41 diagnosed with phonological disorders and 21 with typical phonological development), aged between 4 and 9 years, participated in the study. An identification task was performed in the obstruent class using the speech perception assessment instrument (PERCEFAL). Reaction time, percentage of correct and incorrect answers, and the error pattern were considered in the analysis. **Results:** Regarding auditory–perceptual accuracy, children with phonological disorders had a significantly lower average of correct answers than children with typical phonological development for both obstruent classes. Regarding reaction time, children with phonological disorders showed longer reaction times for the stop class (p≤0.05). In the error pattern analysis, errors involving the articulatory point were the most frequent for both classes and both groups of children.

**Conclusion:**

The presence of phonological disorders implies attenuated perceptual accuracy. The longer reaction time of children with phonological disorders depends on the phonic class.

## INTRODUCTION

According to the DSM-5^(1)^, phonological disorders (PDs) fall under the diagnosis of speech disorders, which are neurodevelopmental disorders diagnosed when speech production does not occur as expected, according to the child's age and stage of development and when the deficiencies are not consequences of physical, structural, neurological or auditory impairment.

Some studies have sought to identify factors that may explain the occurrence of this alteration. Among them, those that point to alterations in the auditory–perceptual domain stand out. This includes changes in auditory processing, assessments of hearing using electrophysiological methods, and how children perform speech perception tasks. These tasks involve distinguishing between sounds and recognizing phonological contrasts^(2-4)^.

In order to find an organic–physiological etiology that could explain the occurrence of PDs, a study^(5)^ investigated the relationship between temporal auditory processing skills and altered distinctive features in cases of PD. Eighteen children aged between 6:0 and 8:0 years, diagnosed with PD, participated in the study. The participants were assessed for their temporal processing skills using the GIN – Gap in Noise Test, FPT – Frequency Pattern Test and DPT – Duration Pattern Test. It was observed that the subjects presented low performance in temporal auditory processing tasks according to the normative standards of the tests.

Considering the studies that evaluated the electrophysiological aspects of hearing in children with PD, one study characterized children with PD according to short-latency auditory evoked potentials (SAEPs) and long-latency auditory evoked potentials (LLAEPs)^(6)^. Twenty-nine children with PD, aged between 5:0 and 7:11 years, participated in the study and underwent SAEP and LLAEP evaluation. Participants were divided into subgroups according to the severity of the PD (i.e., mild, moderate and severe). As a result, it was observed that children with PD presented performances that were not compatible with the normative data for LLAEP. Specifically, the latency values were higher than those indicated in the literature. Furthermore, in general, children with severe PD presented increased latencies for both LLAEPs and SAEPs.

Regarding the behavioral studies on the auditory–perceptual performance of children with PD, it has been proposed that this population could present difficulties in the perceptual domain, specifically with phonetic identification skills. For example, one study found that children with PD had worse accuracy in speech perception in a lexical and phonetic judgment task^(7)^. The authors suggested that sounds produced incorrectly by children with PD would be perceived with less precision than those produced correctly compared to those with typical speech development. The presence of a correlation between the errors presented by both groups of children was also investigated, observing similarity in the phonemes judged as errors in the speech perception and production tasks.

Additionally, two recent studies investigated the auditory–perceptual performance of children with and without PD in phonemic identification tasks. The first study compared the performance of children with and without PD in identifying stops and observed worse auditory–perceptual accuracy in children with PD compared to children without PD, as well as longer reaction times for correct responses^(8)^. The second study compared the auditory–perceptual performance of children with and without PD in identifying fricatives and found differences only in reaction time between the groups and in the error pattern^(9)^. The results showed that children with PD had longer reaction times for correct and incorrect responses. Interestingly, errors involving the place of articulation of fricatives were the most frequent in both groups.

A recent systematic review and meta-analysis investigated whether preschool and school-age children with PD have difficulties in speech perception^(10)^. The systematic search was conducted in eight databases, and the authors registered 71 eligible articles that examined speech perception skills in children with PD. Each study's results and methodological characteristics were reviewed, and each article's reporting of methodological information was assessed. The authors performed a meta-analysis of studies that used the most common type of speech perception assessment task (i.e., lexical and/or phonetic judgment tasks). In 60 of the 71 studies, it was reported that some or all children with PD had difficulties with speech perception. The meta-analysis also showed a significant difference between children with PD and children with typical speech development on lexical and/or phonetic judgment tasks. Overall, the meta-analysis results demonstrate that children with PD have difficulties in speech perception.

Considering the studies reviewed, it can be inferred that the change in speech perception is a critical variable that directly influences the change in speech production. However, few studies have assessed the auditory–perceptual performance of children with PD in tasks of auditory–perceptual identification of phonemic contrasts. In the present study, given the recurrence of production errors involving the phonemes of the obstruent class, we sought to investigate the auditory–perceptual performance in children with and without PD by assessing stop and fricative perception.

Assuming the existence of a possible relationship between PD and perception difficulties, the hypotheses of the present study are: (1) children with PD would present a less accurate and more laborious performance compared to the performance of children with typical phonological development and (2) for both groups, stops and fricatives would differ in terms of auditory–perceptual accuracy, reaction time and error pattern.

The objectives of the present study, therefore, were (1) to compare auditory–perceptual accuracy and reaction time in children with and without PD and (2) to verify whether accuracy, reaction time and error pattern are dependent on the phonetic class (i.e., stops vs. fricatives).

## METHODS

The study was approved by the Research Ethics Committee of the São Paulo State University "Júlio de Mesquita Filho" (Unesp), Marília Campus, under protocol number 67549317.5.0000.5406. The guardians of the participating individuals signed the Free and Informed Consent Form, and there was assent from the participating children. The institution where the collections took place authorized the development of this study.

### Subjects

The study included 62 children aged 4 to 9 years; 41 were diagnosed with PD with speech impairment in both stops and fricatives, among other sound classes (PD), and 21 had typical phonological development (non-PD). The children in the GTF group were recruited from the Supervised Internship of Speech Therapy: Clinical Phonology of the Center for Health Education Studies (CEES), UNESP in Marília. All children in the PD group underwent audiometry when they began their treatment at CEES and did not present any hearing alterations. The data from the PD group were selected from a database related to GPel (Language Study Group – CNPq).

The PD group was composed of children diagnosed with PD with speech production impairment in both stops and fricatives without any associated auditory alteration, and who took the PERCEFAL test^(11)^ for both phonetic classes were selected.

The non-PD group participants were selected according to age and gender, considering the children who composed the PD group. These individuals received a previous hearing screening with normal results and took the PERCEFAL test^(11)^ for both phonetic classes.

Similar parameters were adopted for both groups as a criterion for sample exclusion. Subjects who presented a diagnosis of PD associated with another language disorder, neurological and/or auditory alterations, and those who presented performance below 80% in the word recognition phase of the PERCEFAL test^(11)^ were excluded. The test authors adopted this parameter.

Chart 1 presents the characteristics of the participating subjects.

**Chart 1:** Characterization of subjects

**Group**	**SUBJECT AGE**	**NUMBER OF SUBJECTS**	**SEX**
	4–5:11	3	M
		2	F
	5–6:11	7	M
		2	F
**PD**	6–7:11	11	M
		3	F
	7–8:11	6	M
		2	F
	8–9:11	2	M
		3	F
	4–5:11	2	M
		2	F
	5–6:11	4	M
		1	F
**non-PD**	6–7:11	2	M
		1	F
	7–8:11	4	M
		2	F
	8–9:11	3	M
		0	F

**Caption:** PD = Phonological Disorder Group; non-PD = Group with typical phonological development; M = Male; F = Female

### Materials

Data on the auditory–perceptual performance of obstruents were obtained from the PERCEFAL instrument^(11)^, using the PERCEVAL (Perception Evaluation Auditory & Visual) software.

As reported in previous studies^(8,11-13)^, PERCEFAL is a phonemic identification test, also called a forced-choice test, involving minimum pairs of words, which can be used with children from four years of age. It is a test comprising a subset of four experiments: PERCivogais, PERCocl, PERCifric, and PERCison. In this study, only PERCocl and PERCifric were used.

The test included 30 words in Brazilian Portuguese from each phonetic class (15 contrastive pairs) represented by pictures, totaling 60 stimuli, possibly familiar to the child's vocabulary. Chart 2 shows all the contrastive pairs present in the identification test.

**Chart 2:** Table of contrastive pairs of obstruents

**FRICATIVES**		**STOPS**	
**Contrasts**	**Minimal pairs**	**Contrasts**	**Minimal pairs**
faca – vaca	/f/ - /v/	berço – terço	/b/ - /t/
fanta– santa	/f/ - /s/	bola – cola	/b/ - /k/
forro – zorro	/f/ - /z/	gola – bola	/g/ - /b/
fora – chora	/f/ - /ʃ/	bote – pote	/b/ - /p/
faca – jaca	/f/ - /Ʒ/	bucha – ducha	/b/ - /d/
vela – sela	/v/ - /s/	danço – ganso	/d/ - /g/
cavar – casar	/v/ - /z/	guerra – terra	/g/ - /t/
veia – cheia	/v/ - /ʃ/	pato – gato	/p/ - /g/
vaca – jaca	/v/ - /Ʒ/	pente – dente	/p/ - /d/
caçar – casar	/s/ - /z/	porta – corta	/p/ - /k/
sapa – chapa	/s/ - /ʃ/	tia – dia	/t/ - /d/
selo – gelo	/s/ - /Ʒ/	torta – porta	/t/ - /p/
rosa – rocha	/z/ - /ʃ/	cola – gola	/k/ - /g/
zangada – jangada	/z/ - **/ Ʒ /**	couro – touro	/k/ - /t/
xiz– giz	/ʃ/ - /Ʒ/	fada – faca	/d/ - /k/

The selection of words that make up the instrument was carried out according to the following criteria: they must contrast the phonemes of Brazilian Portuguese (BP) to compose minimum pairs of words, preferably in accented syllables; they must be capable of being represented using pictures; they must preferably be paroxytone nouns and belong to the child's vocabulary^(11)^.

This instrument consists of visual and auditory stimuli. The auditory stimuli include edited audio recordings of the target words produced by a typical BP-speaking adult. On the Other hand, the visual stimuli correspond to images that directly correspond to the target words, i.e., there is a corresponding image for each target word. The images were obtained from the public domain website Google Images^(14)^ The images were cropped and edited to standardize them using the Paint software, thus resulting in the visual stimuli of the experiment^(11,13)^.

### Experimental procedure

The auditory–perceptual test used in this study, PERCEFAL^(11)^, refers to an identification test in which a choice is made based on the sound–picture relationship. The experiment has three stages: word recognition, training phase and test phase.

In the first stage, called the recognition phase, participants are presented with the visual and auditory stimuli present in the test. This process investigates the child's familiarity with the stimuli. If the child did not recognize 80% of the stimuli presented, they were excluded from the sample.

The training phase, which corresponds to the second stage of the test, is performed automatically by the software to ensure that the identification task is understood. To this end, ten stimuli are randomly selected by the software. These stimuli (auditory and visual) are presented almost simultaneously so that immediately after the presentation of the sound stimulus (target word), two figures (corresponding to minimal pairs) are displayed on the computer screen, with only one of them corresponding to the sound stimulus. After the stimuli are presented, the child must choose which picture corresponds by pressing a previously agreed-upon computer key. However, the software does not compute the results in this phase. It is only after the participants have ensured they understand the task that the test phase begins.

The test phase corresponds to the third and final stage of the experiment. The children remained comfortably seated in front of a computer screen with Koss headphones attached to their ears in the acoustic treatment room of the Acoustic and Articulatory Analysis Laboratory of Center for Health Education Studies (CEES) – UNESP Marília.

The children listened individually (with binaural presentation, at an intensity of 50 dB SPL) to one of the words in the minimal pair. Then, they had to decide and indicate which picture corresponded to the word from two pictures displayed on the computer screen by pressing a previously agreed key.

For example, for the stop test, the word "duck" was presented aurally. Then, pictures corresponding to the words "duck" and "cat" were displayed on the computer screen so that the participant could decide and indicate which of the images would correspond to the auditory stimulus presented.

Similarly, in the identification test involving fricatives, for example, the word "knife" was presented aurally and then pictures corresponding to the words "cow" and "knife" were displayed on the computer screen so that the participant could decide and indicate which of the pictures corresponded to the auditory stimulus presented.

Both the presentation time of the auditory and visual stimuli, as well as the response or reaction time of the participants, were automatically measured by the PERCEVAL software.

Each experiment lasted an average of 15 minutes per child, considering the three stages.

### Data analysis

A descriptive and inferential statistical analysis of the data was performed to verify differences in accuracy and reaction time of both groups of children and the effect of the phonic class on auditory–perceptual performance.

To analyze auditory–perceptual accuracy and reaction time (percentages of correct and incorrect responses), the Repeated Measures ANOVA was used, considering the clinical condition of the children (non-PD and PD) as intergroup variables and the phonological class (stop vs. fricative) as intragroup variable. An alpha (α) value equal to or less than 0.05 was established.

The typology of errors presented in the perception test was analyzed to analyze the error pattern, considering all subjects involved in the study (non-PD and PD). The Repeated Measures ANOVA test was used again, considering the phonological class (stop and fricative) as the intergroup variable and the types of errors as intragroup variables: voicing, articulation point, and voicing + articulation point errors.

## RESULTS

Table 1 presents descriptively the mean of the general auditory-perceptual accuracy (% of correct and incorrect answers) and standard deviation of the PD and non-PD groups.

**Table 1:** Distribution of percentage and SD of accuracy and reaction time of children with and without SD

**Group**	**% Fricatives Correct**	**% Stops correct**	**% Fricatives Correct**	**% Stops incorrect**
**PD**	83.94 (11.05)	80.13 (±16.81)	13.06 (±10.75)	15.01 (±13.08)
**non-PD**	87.12 (±10.72)	89.38 (±8.02)	9.77 (±8.04)	8.76 (±5.36)

**Caption:** PD = Phonological disorder group; non-PD = Group with typical phonological development; % = Percentage; ± = Standard deviation

As indicated in Table 1, the Repeated Measures ANOVA showed a significant effect for groups (F(1,60)= 4.11, p= 0.04) but did not show a significant effect for class (F(1,60)= 0.24, p= 0.62), nor for the interaction between group and class (F(1,60)= 3.69, p= 0.59). In particular, the PD group displayed a lower average accuracy (83.94) than the non-PD group (87.12).

Regarding the percentage of errors, the Repeated Measures ANOVA did not show a significant effect for groups (F(1,60)= 3.32, p=0.07), for class (F(1,60)= 0.15, p= 0.69) or for the interaction between group and class (F(1,60)= 1.49, p=0.22). Table 2 shows the mean Reaction Time and standard deviation of the non-PD and PD groups.

**Table 2:** Average reaction time and SD (ms) of children with and without PD

**Group**	**Reaction time for correct responses**		**Reaction time for incorrect responses**	
	**Fricatives**	**Stops**	**Fricatives**	**Stops**
**PD**	1884.5 (±244.19)	2001.7	2174.1	2318.7
		(±295.86)	(±504.11)	(±532.31)
**non-PD**	1883.9 (±525.92)	1799.7	1899.1	1994.9
		(±448.46)	(±737.58)	(±476.25)

**Caption:** PD = Phonological disorder group; non-PD = Group with typical phonological development; SD = Standard Deviation; ± = Standard Deviation; ms = milliseconds

For the data related to the reaction time (RT) of the correct answers, the Repeated Measures ANOVA did not show a significant effect for group (F(1,60)= 1.43, p= 0.23) or class (F(1,60)= 0.13, p= 0.71), but showed a significant effect for the interaction between group and class (F(1,60)= 4.82, p= 0.03), that is, the group that presented the longest reaction time was dependent on the phonic class.

**Figure 1:**
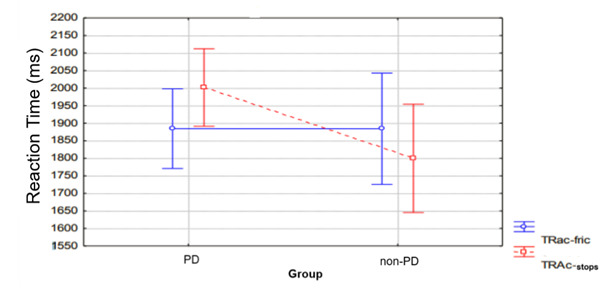
Average reaction time and SD between children with and without PD for correct answers

A post-hoc analysis was then performed using the Fisher t-test, which showed that the difference in RT between classes was only for the PD group. In particular, the PD group presented a higher RT for the stop class than the fricative class, as shown in Figure 1.

**Caption:** PD = Phonological Disorder; non-PD=Group with typical phonological development; ms = milliseconds; TRac-fric = reaction time for correct answers in the fricative class; TRac-stops = reaction time for correct answers in the stop class

For the RT of errors, the Repeated Measures ANOVA showed no main effect for group (F(1,60)= 2.31, p= 0.13), class (F(1,60)= 0.42, p= 0.51) and interaction between group and class (F(1,60)= 1.02, p= 0.31). Table 3 presents the general average (in percentage) of the error pattern concerning the stop and fricative classes.

**Table 3:** Overall average (in percentage) of the error pattern considering both groups (PD and non-PD) in relation to the phoneme classes (stops and fricatives)

**Standard error %**	**Stops- Average**	**Fricatives- Average**	**Total**
Articulatory point	25.86 (90/348)	23.27 (81/348)	49.13 (171/348)
Voicing	13.79 (48/348)	10.05 (35/348)	23.84 (83/348)
Articulatory point + voicing	11.78 (41/348)	15.22 (53/348)	27 (94/348)
Total	51.43 (179/348)	48.54 (169/348)	99.97 (348/348)

**Caption:** PD = Phonological disorder group; non-PD = Group with typical phonological development; % = Percentage

In the analysis of the error pattern, the repeated measures ANOVA did not show a significant effect for class (F(1,60)= 0.01, p= 0.91) or for the interaction between class and type of error (F(1,60)= 2.22, p= 0.11), but there was a significant effect for type of error (F(1,60)= 7.06, p= 0.002).

**Figure 2:**
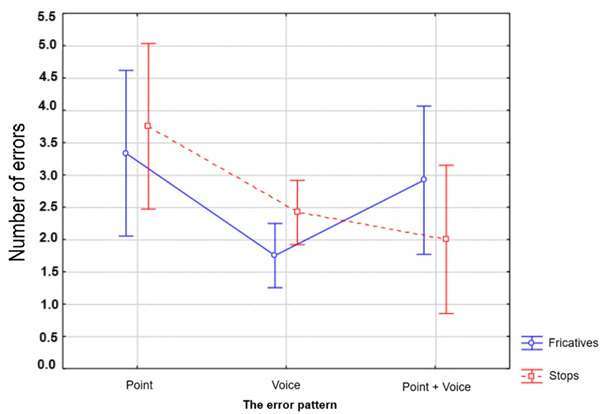
Error pattern in relation to phonological class

Subsequently, the Bonferroni post-hoc test was performed to investigate the difference between the error types. It was observed that articulation point errors differ from voicing and voicing + articulation point errors, as they are the most frequent, as shown in Figure 2.

**Caption:** Point = articulation point; Voice = voicing; Point + Voice = articulation point + voicing

In summary, the PD group presented lower auditory–perceptual accuracy in relation to their age peers with typical phonological development. Furthermore, the PD group was more laborious in identifying the stop class. The phonic class only affected the RT of the PD group. The most frequent error pattern for both groups involved the articulatory point.

## DISCUSSION

The study's objectives were (1) to compare auditory–perceptual accuracy and reaction time in children with and without PDs and (2) to verify whether accuracy, reaction time and error pattern depend on the obstruent phonetic class, i.e., stops vs. fricatives.

It was hypothesized that (1) children with PD would present a less accurate and more laborious performance compared to the performance of children with typical phonological development and (2) for both groups, stops and fricatives would differ in relation to auditory–perceptual accuracy, reaction time and error pattern.

Regarding auditory–perceptual accuracy, the hypothesis was partially confirmed as the groups differed only in the percentage of correct answers, in which children with PD presented a lower percentage of correct answers than children with typical phonological development.

These results corroborate previous studies^(5,6)^, which reported that the lower percentage of correct responses by children with PD is due to their inability to decode and organize auditory stimuli. The findings on auditory–perceptual accuracy also agreed with the results found in other studies^(10,15,16-18)^, which showed that children with PD have less accurate speech perception than children with typical phonological development, which, according to the authors, would be a reflection of a change in the phonological representation of the language.

Regarding the percentage of error results, the PD and non-PD groups did not differ from each other. A possible explanation for the lack of differentiation between the groups is that both groups have children who are in the process of phonological acquisition. Thus, we can infer that difficulties inherent to the language during the phonological acquisition process^(18)^ could be present for both groups, suggesting that "perceptual errors" would be similar to the groups since they present perceptual characteristics of greater difficulty resulting from the language itself.

Regarding reaction time, it was hypothesized that the PD group would be more laborious than the non-PD group. The results partially confirmed this hypothesis, as the groups differed only in the RT of correct responses. These results corroborate results reported in a previous study^(8)^, evidencing greater information processing time by children with PD and, consequently, reflecting the perceptual difficulty presented by the PD group.

Authors report in a study^(19,20)^ that using reaction time is an important form of analysis in identification studies since it reduces uncertainties surrounding results involving phonetic boundaries and perceptual and categorical analyses of speech sounds. Furthermore, the authors state that RT reflects the processing time for decision-making in perceptual tests.

The fact that the PD and non-PD groups did not differentiate themselves by RT in the task of identifying errors may be linked to the perceptual difficulties imposed by the acoustic characteristics of the language segments since the literature reports a gradual auditory–perceptual acquisition as age increases, as well as a hierarchy in acquisition according to the following decreasing order: vowels > sonorants > stops > fricatives^(20)^. Thus, considering the age range of the participants in this study (4 to 9 years and 11 months), it is noted that some of the participants are in the process of phonological acquisition; that is, the perception of phonic contrasts imposes difficulties for both groups of children, which would justify the lack of differentiation in performance by RT considering errors.

Regarding the influence of the phonetic class on the auditory–perceptual performance between the PD and non-PD groups, it was expected that the accuracy, RT and error pattern would depend on the phonetic class. The results only partially confirmed this hypothesis since there was a difference between the phonetic classes only for the reaction time in the PD group.

For the PD group, the stop class presented a higher reaction time than the fricative class. From an acoustic point of view, the stop class is characterized by a long period of silence, corresponding to the blocking of the articulators and a reduced time of acoustic information, that is, from the explosion to the formant transition. Consequently, these acoustic characteristics make the stop class a phonemic class that is more difficult to perceive^(21)^. On the other hand, the fricative class presents a longer time of acoustic information due to the longer blocking of the articulators and friction of the release of air^(22)^, which could facilitate the perception of this class.

The stop and fricative classes did not differ in terms of accuracy or error pattern. Both classes presented a similar number of errors (179 errors for the stop class and 169 for the fricative class) and the same error pattern. The most frequent errors were those involving the articulatory point, corroborating previous studies^(22-24)^, which state that the voicing cue is more robust perceptually, while the articulatory point cue is less salient than the cues that mark voicing and voicing + articulatory point^(25)^.

This result demonstrates that the difficulty in speech perception for children found in the error pattern — articulatory point — does not occur in the degree of constriction of the articulators; that is, the difficulty is not due to the amplitude of the tongue movement but rather in the location of the constriction, which concerns the place where the tongue will be positioned to produce the target sound, which corroborates the studies cited previously^(26,27)^.

In short, it is believed that the findings of the present study (1) provided information regarding the perceptual performance of children with PD and can (2) assist speech-language pathology clinics in analyzing the perceptual performance of individuals with speech–language disorders, as well as (3) consider approaches in phonological rehabilitation that encompass therapeutic stages aimed at the development of phonological perception and production.

We would like to point out as a limitation of this study that neither the severity of the PD nor the PD subtype were considered. Furthermore, the auditory–perceptual investigation was restricted to the class of stops and fricatives. Therefore, for future studies, we suggest auditory–perceptual investigation for the other phonological classes of BP.

## CONCLUSION

Children with PD have worse accuracy regarding the percentage of correct answers and a longer RT for correct answers than those with typical phonological development. It is also worth noting that the stop class presents a longer RT for children with PD and that errors involving the articulatory point were the most frequent for both groups.
