# Supplementary material for: Identification of obstruent contrasts by children with and without phonological disorders
Source: Codas. 2025 Mar 3;37(2):e20240086. doi: 10.1590/2317-1782/e20240086en (PMC11895833; doi:10.1590/2317-1782/e20240086en)
Supplement: Translation

# Translation: Identificação perceptivo-auditiva de contrastes de obstruintes por crianças com e sem Transtorno Fonológico

**DOI:** 10.1590/2317-1782/e20240086pt

**Authors:** Mayara Ferreira de Assis, Elissa Barbi Mouro Pagliari Cremasco, Isabella Rodrigues Domingues, Larissa Cristina Berti

**Affiliations:** 1 Universidade Estadual Paulista “Júlio de Mesquita Filho” – UNESP - São José do Rio Preto (SP), Brasil.; 2 Universidade Estadual Paulista “Júlio de Mesquita Filho” – UNESP - Marília (SP), Brasil.

**Keywords:** Percepção, Avaliação, Transtorno Fonológico, Desenvolvimento Infantil, Inteligibilidade de Fala

## Abstract

**Objetivo:**

(1) comparar a acurácia perceptivo-auditiva e o tempo de reação em crianças com e sem transtorno fonológico na identificação do contraste das obstruintes; (2) verificar se há um efeito da classe fônica (oclusivas vs fricativas) na acurácia, no tempo de reação e no padrão de erro.

**Método:**

Participaram do estudo 62 crianças (41 com diagnóstico de transtorno fonológico e 21 com desenvolvimento fonológico típico), entre 4 a 9 anos de idade. Foi realizada uma tarefa de identificação na classe das obstruintes, a partir do instrumento de avaliação de percepção de fala (PERCEFAL). O tempo de reação, a porcentagem de erros e acertos, bem como o padrão de erro foram considerados na análise.

**Resultados:**

Com relação à acurácia perceptivo-auditiva, as crianças com transtorno fonológico tiveram uma média de acerto significativamente inferior quando comparadas às crianças com desenvolvimento fonológico típico, para ambas as classes. Quanto ao tempo de reação, as crianças com transtorno fonológico apresentaram maior tempo de reação para a classe das oclusivas (p≤0,05). Na análise do padrão de erro, os erros envolvendo o ponto articulatório foram os mais frequentes para ambas as classes e para ambos os grupos de crianças.

**Conclusão:**

A presença do transtorno fonológico implica em menor acurácia perceptual. O maior tempo de reação das crianças com transtorno fonológico é dependente da classe fônica.

## INTRODUÇÃO

Segundo o DSM-5^(1)^, o Transtorno Fonológico (TF) enquadra-se no diagnóstico de Transtorno de Fala, que é um Transtorno do Neurodesenvolvimento, diagnosticado quando a produção da fala não ocorre como esperado, de acordo com a idade e o estágio de desenvolvimento da criança e, ainda, quando as deficiências não são consequências de prejuízo físico, estrutural, neurológico ou auditivo.

Alguns estudos^(2-4)^ têm buscado identificar fatores que possam explicar a ocorrência desta alteração. Dentre eles, destacam-se aqueles que apontam para alterações no domínio perceptivo-auditivo, seja relativo ao processamento auditivo, avaliação eletrofisiológica da audição, seja relativo ao desempenho das crianças em tarefas de percepção da fala, tanto de discriminação auditiva quanto de identificação perceptivo-auditiva de contrastes fonológicos^(2-4)^.

A fim de encontrar uma etiologia orgânico-fisiológica que explicasse a ocorrência do TF, um estudo^(5)^ investigou a relação entre as habilidades de processamento auditivo temporal e os traços distintivos alterados nos casos de transtorno fonológico. Participaram do estudo 18 crianças com idades entre 6:0 e 8:0 anos, com diagnóstico de transtorno fonológico. Os participantes realizaram avaliação das habilidades de processamento temporal por meio dos testes GIN – Gap in Noise Test, TPF – Teste Padrão de Frequência e TPD – Teste Padrão de Duração. Como resultado, observou-se que os sujeitos avaliados apresentaram baixo desempenho nas tarefas de processamento auditivo temporal de acordo com os padrões normativos dos testes. Assim, os autores concluíram que crianças com TF apresentam dificuldade no processamento auditivo temporal.

Considerando a vertente de estudos que avaliaram os aspectos eletrofisiológicos da audição em crianças com TF, cita-se estudo^(6)^ cujo um dos objetivos foi caracterizar crianças com TF quanto à: habilidade dos Potenciais Evocados e Auditivos de curta Latência (PEATE) e de longa latência (PEALL). Participaram do estudo 29 crianças, com idades entre 5:0 anos e 7:11 anos, com TF e submetidas a avaliação do PEATE e do PEALL. Os participantes foram divididos em subgrupos de acordo com o grau de gravidade do TF (leve, moderado e grave). Como resultado, foi observado que as crianças com TF apresentaram desempenho não compatível com os dados normativos para o PEALL, especificamente os valores de latência foram maiores do que o indicado pela literatura. Além disso, de modo geral, as crianças com TF grave apresentaram latências aumentadas tanto para o PEALL quanto para o PEATE. Os autores concluíram, portanto, que crianças com TF apresentam desempenho fora dos padrões normativos nos potenciais evocados e auditivos de curta e longa latência.

No tocante à vertente de estudos comportamentais sobre o desempenho perceptivo-auditivo de crianças com TF, alguns autores^(7-9)^ destacam o fato que esta população poderia apresentar dificuldades no domínio perceptual, especificamente, em habilidade de identificação fônica.

No que se refere ao desempenho de crianças com TF em tarefa de identificação fônica, um estudo^(7)^ constatou pior acurácia da percepção da fala por parte das crianças com TF em tarefa de julgamento lexical e fônico. A partir desses resultados, os autores sugeriram que os sons produzidos incorretamente pelas crianças com TF seriam percebidos com menor precisão do que os sons produzidos corretamente, quando comparados ao grupo de crianças com desenvolvimento típico de fala. Ainda neste estudo, foi investigada a presença de correlação entre os erros apresentados por ambos os grupos de crianças, observando-se semelhança dos fonemas julgados como erro nas tarefas de percepção e produção da fala.

Dois estudos recentes^(8,9)^ também investigaram o desempenho perceptivo-auditivo de crianças com e sem TF em tarefas de identificação fônica. Comparando o desempenho de crianças com e sem TF na identificação de oclusivas, o primeiro estudo^(8)^ registrou pior acurácia perceptivo-auditiva em crianças com TF quando comparadas às crianças sem TF, além de maior tempo de reação em relação aos acertos. O segundo estudo^(9)^, por sua vez, comparou o desempenho perceptivo-auditivo em crianças com e sem TF na identificação das fricativas e encontrou diferenças apenas no tempo de reação entre os grupos, bem como no padrão de erro. Os resultados mostraram que crianças com TF apresentaram maior tempo de reação tanto para os acertos quanto para os erros. Para ambos os grupos, os erros envolvendo o ponto de articulação das fricativas foram os mais frequentes.

Recentemente, foi desenvolvido um estudo^(10)^ que objetivou realizar uma revisão sistemática e meta-análise para investigar se crianças em idade pré-escolar e escolar com TF apresentam dificuldades na percepção da fala. A busca sistemática foi conduzida em 8 bases de dados, os autores registraram 71 artigos elegíveis que examinaram as habilidades de percepção da fala em crianças com TF.

Os resultados e as características metodológicas de cada estudo foram revisados, e o relato das informações metodológicas em cada artigo foi avaliado. Os autores realizaram uma metanálise com estudos que utilizaram o tipo mais comum de tarefa de avaliação da percepção de fala – tarefas de julgamento lexical e/ou fonético. Em 60 dos 71 estudos, foi relatado que algumas ou todas as crianças com TF apresentaram dificuldades com a percepção da fala. A meta-análise mostrou uma diferença significativa entre crianças com TF e crianças com desenvolvimento típico de fala em tarefas de julgamento lexical e/ou fonético. De modo geral, os resultados da meta-análise demonstram que as crianças com TF têm dificuldades na percepção da fala.

Portanto, considerando os estudos resenhados, pode-se depreender que a alteração de percepção da fala é uma variável crítica que influencia diretamente na alteração da produção da fala.

Destaca-se, porém, que ainda são escassos os estudos que investigam o desempenho perceptivo-auditivo em crianças com TF nas tarefas de identificação perceptivo-auditiva de contrastes fonêmicos. Além disso, considerando-se que fricativas e oclusivas compõem uma única classe – as obstruintes -, torna-se interessante investigar o desempenho perceptivo-auditivo em crianças com e sem TF na classe das oclusivas e fricativas conjuntamente, dada a recorrência de erros de produção envolvendo os fonemas dessa classe.

Assumindo a existência de uma possível relação entre TF e dificuldade de percepção, as hipóteses do presente estudo são: (1) crianças com TF apresentariam um desempenho menos acurado e mais laborioso comparativamente ao desempenho das crianças com desenvolvimento fonológico típico; (2) para ambos os grupos oclusivas e fricativas se diferenciariam em relação à acurácia perceptivo-auditiva, tempo de reação e padrão de erro.

Os objetivos do presente estudo, portanto, foram (1) comparar a acurácia perceptivo-auditiva e o tempo de reação em crianças com e sem transtorno fonológico; (2) verificar se a acurácia, o tempo de reação e o padrão de erro são dependentes da classe fônica, ou seja, oclusivas vs fricativas.

## MÉTODO

O estudo teve aprovação do Comitê de Ética em Pesquisa (CEP) da Universidade Estadual Paulista “Júlio de Mesquita Filho” (Unesp), Câmpus de Marília, sob o protocolo nº 67549317.5.0000.5406. Os responsáveis pelos indivíduos participantes assinaram o Termo de Consentimento Livre e Esclarecido e houve assentimento por parte das crianças participantes. A instituição onde ocorreram as coletas autorizou o desenvolvimento deste estudo.

### Sujeitos

Fizeram parte do estudo 62 crianças, de 4 a 9 anos de idade, sendo 41 diagnosticadas com TF com acometimento na produção da fala tanto nas oclusivas quanto nas fricativas, dentre outras classes de sons (GTF), e 21 com desenvolvimento fonológico típico (GT). As crianças do Grupo Transtorno Fonológico (GTF) foram recrutadas do Estágio Supervisionado de Terapia Fonoaudiológica: Fonologia Clínica do Centro de Estudos da Educação da Saúde (CEES), da UNESP Marília. Todas as crianças do GTF passaram por audiometria assim que iniciaram seu atendimento no CEES, não apresentando alterações auditivas.

Os dados do Grupo de crianças com desenvolvimento fonológico típico (GT) foram selecionados de um banco de dados referente ao grupo GPel (Grupo de Estudos sobre a Linguagem – CNPq).

Para composição do GTF, foram selecionadas aquelas crianças que obtiverem o diagnóstico de TF com acometimento na produção de fala tanto nas oclusivas quanto nas fricativas sem qualquer alteração auditiva associada, e as que realizaram o teste PERCEFAL^(11)^ para ambas as classes fônicas.

Para a constituição do GT, as crianças foram selecionadas de acordo com a idade, gênero correspondentes às crianças que compuseram o GTF e que haviam realizado uma triagem auditiva prévia com resultado normal além de terem realizado o teste PERCEFAL^(11)^ para ambas as classes fônicas.

Como critério de exclusão da amostra, adotou-se parâmetros semelhantes para ambos os grupos, foram excluídos os sujeitos que apresentaram diagnóstico de distúrbio fonológico associado à outra alteração de linguagem, alterações neurológicas, e/ou auditivas, e aqueles que apresentaram desempenho inferior a 80% na fase de reconhecimento de palavras do teste PERCEFAL^(11)^, este parâmetro é adotado pelos próprios autores do teste.

O Quadro 1, apresenta as características dos sujeitos participantes.

**Quadro 1:** Caracterização dos sujeitos

**GRUPO**	**IDADE DOS SUJEITOS**	**NÚMERO DE SUJEITOS**	**SEXO**
	4 a 5:11	3	M
		2	F
	5 a 6:11	7	M
		2	F
**GTF**	6 a 7:11	11	M
		3	F
	7 a 8:11	6	M
		2	F
	8 a 9:11	2	M
		3	F
	4 a 5:11	2	M
		2	F
	5 a 6:11	4	M
		1	F
**GT**	6 a 7:11	2	M
		1	F
	7 a 8:11	4	M
		2	F
	8 a 9:11	3	M
		0	F

**Legenda:** GTF = Grupo Transtorno Fonológico; GT = Grupo com desenvolvimento fonológico típico; M= Masculino; F= Feminino

### Material

Os dados referentes ao desempenho perceptivo-auditivo das obstruintes foram obtidos a partir do instrumento PERCEFAL^(11)^, com uso do software PERCEVAL (Perception Evaluation Auditive & Visuelle).

Conforme reportado em estudos anteriores^(8,11-13)^ o PERCEFAL é um teste de identificação fonêmica, também designado teste de escolha forçada, envolvendo pares mínimos de palavras, que pode ser utilizado com crianças a partir dos quatro anos de idade. É um teste composto por um subconjunto de quatro experimentos: PERCivogais, PERCocl, PERCifric, PERCison. Neste estudo, foram utilizados apenas dois experimentos: o PERCocl e o PERCifric.

O teste incluiu 30 palavras de cada classe fônica (15 pares contrastivos) representadas por gravuras, no total de 60 estímulos, possivelmente familiares ao vocabulário infantil. O Quadro 2 apresenta todos os pares contrastivos presentes no teste de identificação.

**Quadro 2:** Quadro de pares contrastivos das obstruintes

**FRICATIVAS**		**OCLUSIVAS**	
**Contrastes**	**Pares mínimos**	**Contrastes**	**Pares mínimos**
faca – vaca	/f/ - /v/	berço – terço	/b/ - /t/
fanta– santa	/f/ - /s/	bola – cola	/b/ - /k/
forro – zorro	/f/ - /z/	gola – bola	/g/ - /b/
fora – chora	/f/ - /ʃ/	bote – pote	/b/ - /p/
faca – jaca	/f/ - /Ʒ/	bucha – ducha	/b/ - /d/
vela – sela	/v/ - /s/	danço – ganso	/d/ - /g/
cavar – casar	/v/ - /z/	guerra – terra	/g/ - /t/
veia – cheia	/v/ - /ʃ/	pato – gato	/p/ - /g/
vaca – jaca	/v/ - /Ʒ/	pente – dente	/p/ - /d/
caçar – casar	/s/ - /z/	porta – corta	/p/ - /k/
sapa – chapa	/s/ - /ʃ/	tia – dia	/t/ - /d/
selo – gelo	/s/ - /Ʒ/	torta – porta	/t/ - /p/
rosa – rocha	/z/ - /ʃ/	cola – gola	/k/ - /g/
zangada - jangada	/z/ - **/ Ʒ /**	couro – touro	/k/ - /t/
xiz– giz	/ʃ/ - /Ʒ/	fada – faca	/d/ - /k/

A seleção das palavras que compõem do instrumento foi realizada de acordo com os seguintes critérios: contrastarem os fonemas do PB, de modo a comporem pares mínimos de palavras, preferencialmente em sílabas acentuadas; serem passíveis de representação por meio de gravuras; serem, preferencialmente, substantivos paroxítonos e pertencerem ao vocabulário infantil^(11)^.

Este instrumento é composto por estímulos visuais e auditivos. Os estímulos auditivos compreendem as edições de gravações em áudio das palavras alvo produzidas por um adulto falante típico do PB, enquanto os estímulos visuais correspondem a imagens que fazem correspondência direta às palavras-alvo, ou seja, para cada palavra-alvo existe uma imagem correspondente. As imagens foram obtidas a partir do Google Imagens^(14)^, de domínio público. Com o auxílio do software Paint, as imagens foram cortadas e editadas, de modo a padronizá-las, resultando, deste modo, nos estímulos visuais do experimento^(11,13)^.

### Procedimento experimental

O teste perceptivo-auditivo utilizado no presente estudo, PERCEFAL^(11)^, se refere a um teste de identificação, em que é feita uma escolha por meio da relação som-gravura. O experimento apresenta 3 etapas: reconhecimento de palavra, fase treino e fase teste.

Na primeira etapa, denominada de fase de reconhecimento, são apresentados aos participantes os estímulos visuais e auditivos presentes no teste. Tal processo investiga a familiaridade da criança com os estímulos. Caso a criança não reconhecesse 80% dos estímulos apresentados, esta era excluída da amostra.

A fase treino, corresponde à segunda etapa do teste, é executada de modo automático pelo software, com intuito de garantir a compreensão da tarefa de identificação. Para tanto, são selecionados pelo software, de modo aleatório, 10 estímulos. Estes estímulos (auditivos e visuais) são apresentados quase simultaneamente, de modo que logo após a apresentação do estímulo sonoro (palavra alvo), duas figuras (correspondentes a pares mínimos) são dispostas na tela do computador, sendo somente uma delas correspondente ao estímulo sonoro. Após a apresentação dos estímulos, a criança deve escolher qual a gravura correspondente, pressionando uma tecla do computador previamente acordada. Contudo, nesta fase, os resultados obtidos não são computados pelo software. Garantida a compreensão da tarefa pelos participantes, inicia-se a fase teste propriamente dita.

A fase teste corresponde à terceira e última etapa do experimento. As crianças continuavam dispostas de modo confortável em frente à tela de um computador com fones Koss acoplados aos seus ouvidos, na sala de tratamento acústico do Laboratório de Análise Articulatória e Acústica (LAAc) da UNESP.

As crianças ouviram individualmente (com apresentação binaural, em uma intensidade de 50 DB NPS), uma das palavras do par mínimo e, em seguida, tiveram que decidir e indicar qual gravura era correspondente, diante de duas gravuras dispostas na tela do computador, apertando uma tecla previamente combinada.

Por exemplo, para o teste das oclusivas foi apresentada auditivamente a palavra “pato” e, em seguida, eram dispostas na tela do computador gravuras correspondentes às palavras “pato” e “gato”, para que o participante decidisse e indicasse qual das gravuras corresponderia ao estímulo auditivo apresentado.

Semelhantemente, no teste de identificação envolvendo as fricativas, por exemplo, foi apresentada auditivamente a palavra “faca” e, em seguida, eram dispostas na tela do computador gravuras correspondentes às palavras “vaca” e “faca”, para que o participante decidisse e indicasse qual das gravuras correspondia ao estímulo auditivo apresentado.

Tanto o tempo de apresentação dos estímulos auditivos e visuais, quanto o tempo de resposta ou reação dos participantes foram mensurados automaticamente pelo software PERCEVAL.

Cada um dos experimentos durou em média 15 minutos por criança, considerando as três etapas.

### Análise dos dados

Realizou-se uma análise estatística descritiva e inferencial dos dados para verificar diferenças em relação à acurácia e ao tempo de reação de ambos os grupos de crianças, bem como para a constatação do efeito da classe fônica sobre o desempenho perceptivo-auditivo.

Para a análise da acurácia perceptivo-auditiva e do tempo de reação (porcentagens de acerto e erros), utilizou-se a ANOVA de Medidas Repetidas, considerando-se como variáveis intergrupos, a condição clínica das crianças (GTF e GT) e como variável intragrupo a classe fônica (oclusiva vs fricativa). Estabeleceu-se um valor de alfa igual ou menor que 0,05.

Para a análise do padrão de erro, foi analisada a tipologia dos erros apresentados no teste de percepção considerando todos os sujeitos envolvidos no estudo (GT e GTF), o teste ANOVA de Medidas Repetidas foi mais uma vez utilizado, considerando-se como variável intergrupo a classe fonológica (fricativa e oclusiva), e como variáveis intragrupos os tipos de erros, sendo eles: erros de vozeamento, erros de ponto articulatório e erros de vozeamento + ponto articulatório.

## RESULTADOS

A Tabela 1, apresenta descritivamente a média da acurácia perceptivo- auditiva geral (% de erros e acertos) e desvio padrão dos grupos GTF (grupo transtorno fonológico) e GT (grupo com desenvolvimento fonológico típico).

**Tabela 1:** Distribuição da porcentagem e DP da acurácia e tempo de reação das crianças com e sem TF

**Grupo**	**% Acertos fricativas**	**% Acertos oclusivas**	**% Erros fricativas**	**% Erros oclusivas**
**GTF**	83,94 (11,05)	80,13 (±16,81)	13,06 (±10,75)	15,01 (±13,08)
**GT**	87,12 (±10,72)	89,38 (±8,02)	9,77 (±8,04)	8,76 (±5,36)

**Legenda: G**TF = Grupo transtorno fonológico; GT = Grupo com desenvolvimento fonológico típico; % = Porcentagem; ± = Desvio Padrão

Conforme indica a Tabela 1, a ANOVA de Medidas Repetidas mostrou efeito significativo para grupos (F(1,60)= 4,11, p= 0,04), mas não mostrou efeito significativo para classe (F(1,60)= 0,24, p= 0,62), nem para a interação entre grupo e classe (F(1,60)= 3,69, p= 0,59). Particularmente as crianças com TF (GTF) tiveram uma média de acerto inferior (83,94) do que as crianças com desenvolvimento fonológico típico (GT) (87,12).

No tocante à porcentagem de erros, a ANOVA de Medidas Repetidas não mostrou efeito significativo para grupos (F(1,60)= 3,32, p=0,07), para classe (F(1,60)= 0,15, p= 0,69) e nem para a interação entre grupo e classe (F(1,60)= 1,49, p=0,22).

A Tabela 2, apresenta a média do Tempo de Reação e desvio padrão dos grupos GT e GTF.

**Tabela 2:** Tempo de Reação (ms) e DP das crianças com e sem TF

**Grupo**	**Tempo de reação para os acertos**		**Tempo de reação para os erros**	
	**Fricativas**	**Oclusivas**	**Fricativas**	**Oclusivas**
**GTF**	1884,5 (±244,19)	2001,7	2174,1	2318,7
		(±295,86)	(±504,11)	(±532,31)
**GT**	1883,9 (±525,92)	1799,7	1899,1	1994,9
		(±448,46)	(±737,58)	(±476,25)

**Legenda:** GTF = Grupo transtorno fonológico ; GT = Grupo com desenvolvimento fonológico típico; DP = Desvio Padrão; ± = Desvio Padrão; ms = milissegundos

Para os dados referentes ao tempo de reação (TR) dos acertos, a ANOVA de Medidas Repetidas não mostrou efeito significativo para grupo (F(1,60)= 1,43, p= 0,23) e nem para classe (F(1,60)= 0,13, p= 0,71), mas mostrou efeito significativo para a interação entre grupo e classe (F(1,60)= 4,82, p= 0,03), ou seja, o grupo que apresentou maior tempo de reação foi dependente da classe fônica.

Foi feita, então, a análise Post-Hoc a partir do teste T-Fisher, na qual se observou que a diferença do TR entre as classes foi apenas para o GTF. Particularmente, as crianças com TF (GTF) apresentaram maior TR para a classe das oclusivas comparativamente à classe das fricativas, conforme ilustra a Figura 1.

**Figura 1:** Tempo de Reação entre crianças com e sem TF para os acertos

**Legenda:** TF = Transtorno Fonológico; T = Típicos; ms = milissegundos; TRacfric = tempo de reação para os acertos classe fricativas; TRac-ocl = tempo de reação para os acertos classe oclusivas

Para o TR dos erros, a ANOVA de Medidas Repetidas não mostrou efeito principal para grupo (F(1,60)= 2,31, p= 0,13), classe (F(1,60)= 0,42, p= 0,51) e interação entre grupo e classe (F(1,60)= 1,02, p= 0,31).

A Tabela 3, apresenta a média geral (em porcentagem) do padrão de erro em relação às classes da oclusiva e fricativa.

**Tabela 3:** Média geral (em porcentagem) do padrão de erro considerando ambos os grupos (GTF e GT) em relação às classes de fonemas (oclusiva e fricativa)

**Padrão de erro %**	**Oclusiva- Média**	**Fricativa- Média**	**Total**
Ponto articulatório	25,86 (90/348)	23,27 (81/348)	49,13 (171/348)
Vozeamento	13,79 (48/348)	10,05 (35/348)	23,84 (83/348)
Ponto articulatório + vozeamento	11,78 (41/348)	15,22 (53/348)	27 (94/348)
Total	51,43 (179/348)	48,54 (169/348)	99,97 (348/348)

**Legenda:** GTF= Grupo transtorno fonológico; GT= Grupo com desenvolvimento fonológico típico; % = Porcentagem.

Na análise do padrão de erros, a ANOVA de medidas repetidas não mostrou efeito significativo para classe (F(1,60)= 0,01, p= 0,91) e para interação entre classe e tipo de erro (F(1,60)= 2,22, p= 0,11), mas houve efeito significativo para tipo de erro (F(1,60)= 7,06, p= 0,002).

Posteriormente, realizou-se o teste Post-Hoc de Bonferroni, para investigar a diferença entre os tipos de erro. Observou-se que erros de ponto articulatório se diferenciam de erros de vozeamento e vozeamento + ponto articulatório, por serem os mais frequentes, como mostra a Figura 2.

**Figura 2:** Padrão de erros em relação à classe fonológica

**Legenda:** Ponto= ponto articulatório; Voz= vozeamento; Ponto +Voz = ponto articulatório + vozeamento

Em síntese, o grupo de crianças com transtorno fonológico (GTF) apresentaram menor acurácia perceptivo-auditiva em relação aos seus pares etários com desenvolvimento fonológico típico. Além disso, o GTF foi mais laborioso na tarefa de identificação da classe das oclusivas. A classe fônica teve efeito somente para o GTF quanto ao tempo de reação. Para ambos os grupos (GTF e GT), o padrão de erro mais frequente foram os que envolveram o ponto articulatório.

## DISCUSSÃO

O estudo apresentado teve como objetivos (1) comparar a acurácia perceptivo-auditiva e o tempo de reação em crianças com e sem transtorno fonológico; (2) verificar se a acurácia, o tempo de reação e o padrão de erro são dependentes da classe fônica, ou seja, oclusivas vs fricativas.

Hipotetizou-se que (1) crianças com TF apresentariam um desempenho menos acurado e mais laborioso comparativamente ao desempenho das crianças com desenvolvimento fonológico típico; (2) para ambos os grupos oclusivas e fricativas se diferenciariam em relação à acurácia perceptivo-auditiva, tempo de reação e padrão de erro.

Em relação à acurácia perceptivo-auditiva, a hipótese foi confirmada parcialmente à medida que os grupos se diferenciaram apenas pela porcentagem de acertos em que as crianças com TF apresentaram menor porcentagem de acerto do que crianças com desenvolvimento fonológico típico.

Tais resultados corroboram estudos anteriores^(5,6)^ os quais reportaram que a menor porcentagem de acertos, por parte das crianças com TF, se deve à inabilidade de decodificação e organização dos estímulos auditivos. Os achados sobre a acurácia perceptivo-auditiva concordaram, ainda, com os resultados encontrados em outros trabalhos^(10,15,16-18)^, os quais reportam que crianças com TF apresentam a percepção de fala menos acurada do que as crianças com desenvolvimento fonológico típico o que segundo os autores seria reflexo de uma alteração na representação fonológica da língua.

No que se refere ao resultado referente à porcentagem de erros, os grupos (GTF e GT) não se diferenciaram entre si. Uma possível hipótese explicativa para a não diferenciação entre os grupos é que ambos os grupos possuem crianças que estão em processo de aquisição fonológica. Deste modo, podemos inferir que para ambos os grupos dificuldades inerente à língua durante o processo de aquisição fonológica^(18)^ poderiam se apresentar, sugerindo que os “erros perceptuais” se mostrariam de modo semelhante aos grupos pelo fato de apresentarem características perceptuais de maior dificuldade decorrentes da própria língua.

No tocante ao tempo de Reação, hipotetizou-se que o GTF seriam mais laboriosos do que o GT. Os resultados obtidos confirmaram parcialmente esta hipótese, na medida em que os grupos se diferenciam apenas no TR dos acertos. Esses resultados corroboram resultados reportados em estudo prévio^(8)^, evidenciando maior tempo de processamento da informação por parte das crianças com TF e, consequentemente, refletindo dificuldade perceptual apresentada pelo GTF.

Autores reportam em estudo^(19,20)^ que a utilização do TR é uma importante forma de análise em estudos de identificação, uma vez que diminui as incertezas em torno dos resultados envolvendo fronteiras fonéticas, análises perceptuais e categóricas dos sons da fala. Além disso, os autores afirmam que o TR reflete o tempo de processamento para a tomada de decisão em testes perceptuais.

O fato de os grupos (GTF e GT) não terem se diferenciado pelo TR na tarefa de identificação para os erros, pode estar vinculado às dificuldades perceptuais impostas pelas características acústicas dos segmentos da língua, já que, a literatura reporta uma aquisição perceptivo-auditiva gradual conforme o aumento da idade, como também uma hierarquia na aquisição de acordo com a seguinte ordem decrescente: vogais>sonorantes>oclusivas>fricativas^(20)^. Assim, considerando a faixa etária dos participantes deste estudo (4 a 9 anos e 11 meses) nota-se que parte dos participantes está em processo de aquisição fonológica, ou seja, a percepção dos contrastes fônicos impõe dificuldade para ambos os grupos de crianças (GTF e GT), o que justificaria a não diferenciação de desempenho pelo TR considerando os erros.

No que se refere à influência da classe fônica no desempenho perceptivo-auditivo entre os grupos GTF e GT, esperava-se que a acurácia, o TR e o padrão de erro fossem dependentes da classe fônica. Os resultados obtidos confirmaram apenas parcialmente esta hipótese, uma vez que houve diferença entre as classes fônicas apenas para o TR no GTF.

Para o GTF, a classe das oclusivas apresentou maior TR comparada à classe das fricativas. No ponto de vista acústico, a classe das oclusivas caracteriza-se por grande período de silêncio, correspondente ao bloqueio dos articuladores e tempo reduzido de informação acústica, ou seja, da explosão à transição formântica. Consequentemente, essas características acústicas torna a classe das oclusivas uma classe fonêmica mais difícil de ser percebida^(21)^. Por outro lado, a classe das fricativas apresenta maior tempo de informação acústica, por ter maior tempo de bloqueio dos articuladores e fricção da soltura do ar^(22)^, tais características poderia facilitar a percepção desta classe.

Em relação à acurácia e ao padrão de erro, as classes das oclusivas e fricativas não se diferenciaram, ou seja, ambas as classes apresentaram número de erros semelhantes (um total de 179 erros para a classe das oclusivas e 169 erros para a classe das fricativas) e o mesmo padrão de erros. Os erros mais frequentes foram aqueles envolvendo o ponto articulatório, corroborando com os estudos anteriores^(22-24)^, os quais citam que a pista de vozeamento é mais robusta perceptualmente; enquanto a pista de ponto articulatório é menos saliente do que as pistas que marcam vozeamento e vozeamento + ponto articulatório^(25)^.

Esse resultado demonstra que a dificuldade de percepção de fala para as crianças encontrada no padrão de erro –ponto articulatório- não se dá no grau da constrição dos articuladores, ou seja, a dificuldade não é por conta da amplitude do movimento de língua, mas sim no local da constrição, que diz respeito ao local em que a língua irá se posicionar para realizar o som-alvo, o que corrobora os estudos citados anteriormente^(26,27)^.

Em suma, acredita-se que os achados do presente estudo (1) forneceu informações a respeito do desempenho perceptual de crianças com TF e pode (2) auxiliar a clínica fonoaudiológica na análise do desempenho perceptivo de indivíduos com alterações fonoaudiológicas bem como (3) considerar abordagens na reabilitação fonológica que abranjam etapas terapêuticas visando o desenvolvimento de percepção e produção fonológica.

Alertamos como limitação deste estudo o fato de não ser considerado a gravidade do TF, nem mesmo o subtipo de TF. Além disso, a investigação perceptiva-auditiva foi restrita à classe das oclusivas e fricativas. Desta forma, para estudos futuros sugere-se, a investigação perceptiva-auditiva para as demais classes fonológicas do PB.

## CONCLUSÃO

As crianças com TF apresentam pior acurácia em relação à porcentagem de acerto e, ainda, maior tempo de resposta referente aos acertos, quando comparadas ao grupo de crianças com desenvolvimento fonológico típico. Vale, ainda, ressaltar que a classe das oclusivas apresenta maior tempo de reação para as crianças com TF, e que erros envolvendo o ponto articulatório foram os mais frequentes para ambos os grupos.
